# Sustainable plant-based ingredients as wheat flour substitutes in bread making

**DOI:** 10.1038/s41538-022-00163-1

**Published:** 2022-10-28

**Authors:** Yaqin Wang, Ching Jian

**Affiliations:** 1 College of Food Science and Technology, Nanjing Agricultural University, Nanjing, People’s Republic of China; 2 Department of Food and Nutrition, University of Helsinki, Helsinki, Finland; 3 Human Microbiome Research Program, Faculty of Medicine, University of Helsinki, Helsinki, Finland

**Keywords:** Microbiology, Sustainability, Developing world

## Abstract

Bread as a staple food has been predominantly prepared from refined wheat flour. The world’s demand for food is rising with increased bread consumption in developing countries where climate conditions are unsuitable for wheat cultivation. This reliance on wheat increases the vulnerability to wheat supply shocks caused by force majeure or man-made events, in addition to negative environmental and health consequences. In this review, we discuss the contribution to the sustainability of food systems by partially replacing wheat flour with various types of plant ingredients in bread making, also known as composite bread. The sustainable sources of non-wheat flours, their example use in bread making and potential health and nutritional benefits are summarized. Non-wheat flours pose techno-functional challenges due to significantly different properties of their proteins compared to wheat gluten, and they often contain off-favor compounds that altogether limit the consumer acceptability of final bread products. Therefore, we detail recent advances in processing strategies to improve the sensory and nutritional profiles of composite bread. A special focus is laid on fermentation, for its accessibility and versatility to apply to different ingredients and scenarios. Finally, we outline research needs that require the synergism between sustainability science, human nutrition, microbiomics and food science.

## Introduction

Bread is known as one of the most ancient foods and widely consumed in all its various forms by humanity. Wheat grains contain unique gluten proteins that impart viscoelastic properties to dough required for leavened bread making^1^. In the Western world, refined wheat flour has been the standard raw material for bread production^2^. The consumption of refined wheat bread has been increasing rapidly in the developing countries due to urbanization and industrialization^3^, and is associated with the burden of non-communicable diseases^4^. Meanwhile, ~3 billion people, most of which are in Asia and Africa, could not afford a healthy diet in the pre-pandemic period^5^. A primary driver of this increasingly dire situation is the double burden of climate shocks and violent conflict in areas that are already food insecure^6^. Africa imports over 60% of its wheat flour needs, of which a significant proportion depends on the wheat production in Russia and Ukraine^7^. Therefore, global hunger is projected to rise radically following the fallout of the COVID-19 pandemic and the large-scale military conflict between Russia and Ukraine. On the other hand, in developed countries, consumers are increasingly aware of the health and environmental benefits of bread products produced partially using non-wheat ingredients, which are thought to be low in glycemic index (GI; a value used to measure how much specific foods increase blood sugar (glucose) levels), rich in protein, dietary fiber and various bioactive compounds^8–11^. They are also supposedly lower in the carbon and water footprint compared to refined wheat bread, contributing to environmental sustainability^12,13^.

**Fig. 1:**
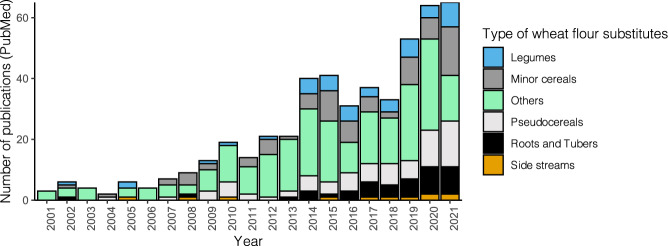
Number of publications per year in the PubMed database related to composite bread over the past 20 years (2001–2021). The search was conducted using the following key words: (bread [Title/Abstract]) AND ((“composite flour”) OR (“non-wheat”) OR ((legum*) AND (flour)) OR ((pulse*) AND (flour)) OR ((root*) AND (flour)) OR ((tuber*) AND (flour))OR ((barley) AND (flour)) OR ((sorghum) AND (flour)) OR ((amaranth) AND (flour)) OR ((quinoa) AND (flour)) OR ((BSG) AND (flour)) OR ((buckwheat) AND (flour)) OR ((oat) AND (flour)) OR ((millet) AND (flour)) OR ((oilseed) AND (flour)) OR ((cassava) AND (flour)) OR ((yam) AND (flour)) OR ((potato) AND (flour)) OR ((side stream) AND (flour))).

The concept of reducing wheat importation by replacing part of it with indigenous crops in food production in developing countries dates back to the 1960s, which was envisioned to increase food security in vulnerable regions. In the context of bread making, the bread produced by using a combination of wheat and wheat flour substitutes has been described as composite bread. Despite the growing interest in composite bread in recent years, the development of composite bread has been primarily limited to home baking and its associated research is relatively scant (Fig. 1). Among other factors, low consumer acceptability and unfamiliarity with the benefits of composite bread represent major obstacles^14,15^. Recently, the processing strategies for improving the quality of composite bread have gained increasing interest, and sustainable bread production becomes imperative in the post-crisis era. Therefore, in this review, we discuss the significance of wheat flour substitutes in sustainable bread making. We then summarize source materials that have shown potential as wheat flour substitutes and recent advances in the processing approaches utilized to improve their techno-functionality, sensory characteristics, and nutritional values. This review also attempts to give directions for future studies aiming to develop composite bread using novel ingredients. As transformations toward more sustainable and climate-resilient food systems require a major shift to plant-based diets in most parts of the world^16^, we focus on major climate-resilient crops (CRCs) and their processing side-streams^3^. CRCs are sometimes described as neglected and underutilized crop species (NUS) depending on different regions^17^. To increase the applicability of this review, the processing strategies discussed herein have all been applied to different types of composite bread and have shown positive effects on bread quality.

## Wheat flour substitutes in sustainable breadmaking: why and how?

### The influence of bread production in sustainable food systems

Achieving sustainability in food production and trade is one of humanity’s contemporary challenges and requires a holistic approach. A sustainable food system is described by the Food and Agriculture Organization of the United Nations (FAO) as a framework encompassing a myriad of elements, including economic, social, and environmental dimensions, which delivers food security and nutrition for all as well as for future generations. Resilience and sustainability are complementary concepts: a resilient food system is able to provide sufficient, appropriate and accessible food to all, in the face of various and even unforeseen disturbances^18^. Bread is a worldwide staple food and thus has a substantial influence on the sustainability of food systems, especially in terms of environmental impact, food security and food system resilience, and human health. These important elements that contribute to a sustainable food system have been increasingly challenged by reliance on wheat flour in bread production.

### Negative consequences of dependence on wheat grains

**Fig. 2:**
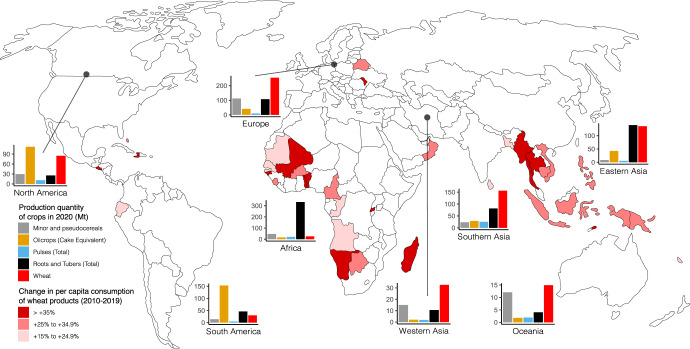
Global production of selected climate-resilient crops (CRCs) and wheat in 2020, and countries with increased consumption of wheat products between 2010 and 2019. The production quantity of five crop types mentioned in the present review is presented as bar charts. Crops belonging to oil crops, pulses, roots and tubers are grouped according to FAO’s categories, whereas minor and pseudocereals include barley, buckwheat, millet, oats, quinoa, and sorghum. The unit of production quantity is million tonnes (Mt). The production quantity of individual countries is aggregated to the indicated regional level according to FAO’s categories. The consumption data is expressed as relative change. Data from FAO^5^ (https://www.fao.org/faostat/en/#data, accessed 30/04/2022).

Global annual average production of wheat was 750 million tonnes (Mt) over the 5-year period from 2015 to 2020^5^ and depends on a few breadbaskets. China is the leading wheat producer, accounting for 17.6% of the world total wheat production in 2020, whereas the other top producers e.g., India, Russian Federation, the United States of America, Canada, and France account for 40.5%^5^. It is estimated that wheat production should increase by 87 Mt to 840 Mt by 2030 to meet future food demands^19^. The drastic increase in wheat cultivation has intensified the need for sustainable food production. On the other hand, wheat consumption is expected to increase by 12% by 2030, where more than two-thirds are used for food^19^. The adoption of western lifestyle and diet due to urbanization and industrialization in developing countries is the major driving force for increasing wheat demand^20^ (Fig. 2). The increase in wheat consumption is especially concentrated in Africa and the middle East/Western Asia, most of which are beyond the regions of wheat production and heavily rely on wheat imports that are susceptible to systemic disruptions^21^ (Fig. 2). The COVID-19 pandemic and the recent Russian-Ukraine armed conflict, which both have long-lasting ramifications in wheat production and supply chain disruptions, have added more pressure on food system resilience with negative consequences for food security in these vulnerable regions in the years to come. The current share of global wheat importation by Africa and the Middle East/Western Asia is ca. 45% and is predicted to rise due to increased adverse weather events (e.g., rising temperatures and declining rainfall) accentuated by climate change. Climate change causes volatility in crop yields and fluctuations in wheat prices, leading to uncertainty about future wheat availability in the vulnerable regions^22^. Moreover, wheat, rice, and maize are responsible for up to 60% of nutrient runoff globally^23^. It has been estimated that over 50% of the environmental impact of producing an 800-g loaf of wheat bread arises directly from wheat cultivation, with the use of ammonium nitrate fertilizer alone accounting for around 40%^24^. This negative environmental impact perpetuates a vicious cycle, increasing the fragility of the global food system.

Low- and middle-income countries now experience the highest prevalence and mortality rates of cardiovascular disease^25^. The increased use of refined wheat flour in current breadmaking practices has been associated with a higher risk of mortality and major cardiovascular disease events^4^. On the other hand, the consumption of bread made of whole-grain cereals or enriched with bioactive compounds is generally recognized as health-promoting^26^, and has been explored as an approach to improve cardiometabolic profile^27^. Taken together, sustainable bread production becomes imperative in the challenging time. This will require a fundamental transformation of current practices that rely predominantly on wheat grains, preferentially in ways that prioritize the needs of vulnerable regions as the impacts of food insecurity are highest in these regions^6^.

### Substitution of wheat flour as a solution to sustainable bread production

**Fig. 3:**
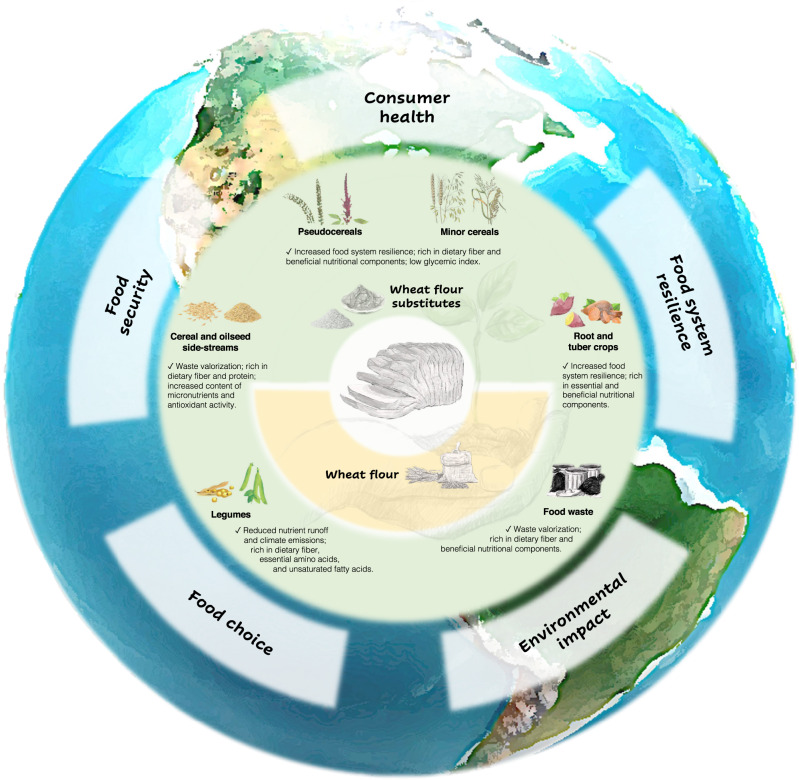
Overview of wheat flour substitutes in relation to sustainable bread production.

Diversification of plant-based food sources is necessary to improve the sustainability in global food systems. In addition to reduced environmental impact, utilization of indigenous grain crops in industrial processes contributes to local economic development. The shorter food supply chains provide easier access to healthy and affordable food in crisis situations, promoting food system resilience. Moreover, unlike refined wheat flour, many non-wheat cereals and legumes possess dense nutritional composition and a range of health-promoting bioactive compounds and dietary fibers with diverse structures^8,28^. For instance, wheat contains lower concentrations of β-glucan that differs from oat and barley β-glucans in molecular structure^29^. Oat and barley β-glucans are relatively more soluble and shown to maintain gut health by various mechanisms, including modulation of the gut microbiota (i.e., collection of host-associated microbes living in the gut)^30^. A major function of the gut microbiota is to process food ingredients, mostly non-digestible carbohydrates, so that the foods of their biotransformation can be utilized by the host. Due to its considerable contribution to digestion, immune and metabolic homeostasis, the gut microbiota has been associated with various intestinal and extraintestinal diseases^31^. Hence, inclusion of minor cereals and pseudocereals in wheat-based foods diversifies dietary fiber sources, which is conductive to gut and metabolic health^32^. On the other hand, agricultural food wastes and byproducts, produced in huge amounts in industrialized countries, represent underutilized and virtually unlimited sources of bioactive compounds, including dietary fibers. The Western diet is deprived of dietary fibers, many of which (e.g., β-glucan) are considered as prebiotics that are defined as “substrates selectively utilized by host-associated microbes conferring a health benefit”^33^. Waste valorization in food production can be thus leveraged to correct our fiber-impoverished modern diet^34^. Therefore, improved food security and food system resilience, human health, and reduced environmental cost can be integrated into a common framework of sustainable bread production through the substitution of wheat flour (Fig. 3).

While substituting wheat flour with non-wheat alternatives in breadmaking has been advocated for over five decades^35^, the success is limited in low- and middle-income countries chiefly due to the inferior technological quality and sensory attributes to refined wheat bread. Counterintuitively, relatively little systemic research has been conducted for improving breadmaking potential of non-wheat cereals and crops to date^9^. Transition to sustainable food products requires shifts in food choices that are heavily influenced by hedonic and sensory aspects of taste and texture/mouthfeel, which is best exemplified by the 2012 Nigerian policy stipulating the inclusion of 40% cassava in composite flour in bakery products^36^. According to recent local surveys, this initiative has been largely thwarted by low acceptability of cassava composite bread, high cost of bread improvers, lack of processing technologies among bakers and ignorance among consumers regarding the choices and benefits of the composite bread^14,15^.

## Sources of wheat flour substitutes in bread making

### Legumes, cereal side-streams, and oilseed meal-based ingredients

Rapid population growth, increasing incomes, and accelerated urbanization have led to an increased demand for animal-based proteins globally, particularly in developing countries, which comes at the expense of environment and public health^37^. Plant-based proteins are considered more environmentally friendly than animal proteins due to their lower carbon footprint, land use, and water use^38^. Moreover, plant proteins can be recovered from byproducts (e.g., bran fractions and oilseed press cakes) and enter back into the food production chain, improving sustainability and thus building a circular economy. From a nutritional point of view, incorporating plant protein ingredients in wheat bread contributes to a higher protein intake with a better amino acid profile. For instance, symptoms of tryptophan deficiency, including reduced growth, impaired bone development, and neurological abnormalities, often occur in parts of sub-Saharan Africa where maize, known to be deficient in tryptophan, is the staple food^39^. Some legumes and protein-rich products made from rapeseed are natural sources of tryptophan, provided the inherent antinutritive factors (e.g., protease inhibitors) are properly processed^40^. Various forms and sources of plant proteins have been studied as wheat alternatives that differ in protein content and functional properties as listed in Supplementary Table 1.

#### Legume-based

Leguminosae family comprises 800 genera and 20,000 species grown worldwide^41^. Legumes are well known for their nitrogen fixing ability that reduces the emission of greenhouse gases in agro-ecosystems^42^. They also have low carbon and water footprints; food legumes occupy a minimal part of arable land^42^. The consumption of legumes has been suggested to provide health benefits via their antioxidant activity, blood pressure lowering, hypoglycemic, hypocholesterolemic, antiatherogenic, anticarcinogenic, and prebiotic properties^43^. Legumes are rich in dietary fiber (8–28 g/100 g) and protein (21–37 g/100 g). According to the European Union (EU) regulation (EC) No 1924/2006 (lastly amended by Regulation (EU) No 1047/2012), a claim that a food product is “high in protein” can be made when ≥20% of the energy value is provided by protein, and a claim “high in fiber” or “source of fiber” can be made when the product contains at least 6 g/100 g or 3 g/100 g fiber, respectively. Proteins sourced from legumes are categorized as incomplete proteins, since they tend to be low in the essential amino acids such as methionine and cysteine. Complete proteins from legume-based foods can be complemented by other crops such as cereals^8^, making them attractive ingredients for composite bread. Composite breads using various types of legume flour are among the most studied wheat flour substitutes (Fig. 1). They can be integrated in bread formulations as either flours or protein isolates (protein content >90%) or concentrates (protein content 60–75%). Findings from our recent studies and previous research have demonstrated that partial substitution of wheat flour with legumes in bread making confers the bakery products with a better amino acid profile, specifically by complementing the deficiencies of lysine and threonine in wheat and inadequate sulfur amino acids in legumes, fulfilling the nutrition claims as “high in protein”^44–46^. In addition, replacing wheat flour with legumes contributes to higher levels of unsaturated fatty acids (e.g., linoleic acid and α-linolenic acid) and bioactive compounds (e.g., vitamins, minerals, total phenolic compounds, and flavonoids) that have been shown to be beneficial for metabolic health^47–50^. Several leguminous plants native to developing countries have been formulated to produce protein-rich composite bread, suggesting the versatility of legume-based wheat flour substitutes that can be adapted according to local cultivars and needs. For example, Erukainure et al. substituted wheat with 15% of bambara flour derived from bambara nut, a grain legume crop indigenous to sub-Saharan Africa^51^. While the inclusion of bambara decreased the loaf volume of bread, the composite bread had comparable perceived taste and aroma compared with wheat bread. Gonzales-Barron et al. replaced wheat flour with up to 15% of mesquite flour made from *Prosopis pallida* (mesquite), native to Peru, to produce a bread product with an enhanced nutritional profile^49^.

#### Cereal side-streams

Wheat, rice, and maize are the most economically important food crops, providing over 50% of the world’s calorie intake^19^. Their bran fractions generated during the milling process are currently utilized mainly as animal feed and in biofuel production^52,53^. Wheat bran and rice bran have a global annual production volume of ~130 and 76 Mt per year, respectively, of which the protein content is 10–25%^52,54^. In other words, the wheat and rice side-streams contain up to 30 Mt of proteins that have largely gone unused for human consumption. Thus, the exploitation of side-streams as food ingredients is expected to improve the sustainability of cereal production, generate indirect income, and provide novel healthy food options for consumers^52^. For example, in the study by Arte et al. a “high in protein” wheat bread was formulated by adding 12.2% (flour weight) of wheat bran protein isolates^53^. In other studies, rice bran protein concentrate was added at up to 15% (flour weight), which significantly increased the protein and fiber content and antioxidant activity of wheat bread^55–57^.

#### Oilseed side-streams

The global oilseed production in 2020/2021 was around 600 Mt;^58^ its byproducts, oilseed press cakes (also known as oilseed meals), are mainly used as feed for livestock, fertilizer, or soil compost^59^, but represent untapped sources of proteins, unsaturated fatty acids (omega-3 and omega-6 fatty acids), and bioactive compounds (e.g., phenolic compounds, carotenoids, lignans, tocopherols, and phytosterols) as human food. Oilseed press cakes can be formulated into flour (protein content <65%), protein concentrates (65–90%), and protein isolates (>90%) by several methods, such as alkaline or acid extraction assisted by salt and enzymes, solvent extraction, or air classification^60,61^. The nutrient recovery from oilseed side-streams is a sustainable and feasible approach in waste management, especially in developed countries. In this respect, attempts have been made to utilize oilseed cakes for the fortification of bread products. Pojić et al. used hempseed cake flour to replace 20% of wheat flour, leading to an increased protein content and micronutrients (e.g., iron), and a decreased starch content of the bread^62^. Similarly, Mohammed et al. reported that the addition of 9% (flour weight) sunflower cake protein isolate to wheat bread formulations resulted in higher protein and amino acid contents, particularly in terms of the essential amino acids^63^. Sanmartin et al. showed that wheat bread enriched with 10% flaxseed cake flour exhibited significantly higher levels of unsaturated fatty acids, total phenols and flavonoids, and the resultant antioxidant activity^64^. In our recent study^65^, rapeseed cake protein concentrate and isolate were formulated into wheat bread at 20 and 10%, respectively, to obtain a bread product fulfilling the EU nutrition claims as “high in protein” and “source of fiber”. By integrating fermentation, the composite breads also have improved amino acid profiles, in particular tryptophan.

### Minor cereals, pseudocereals, and root flours

Minor cereals (e.g., sorghum, millets, barley, and oats), pseudocereals (e.g., quinoa, buckwheat, and amaranth), and root and tuber crops (cassava, sweet potato, and yams), many of which are indigenous crops in vulnerable regions, play an important role in the small-scale farming systems. These crops have remained largely neglected in commercial food production due to the lack of processing technologies, and therefore the consumption is restricted mainly to their growing regions. On the other hand, findings from recent studies support the prebiotic properties and beneficial metabolic effects of millets^66,67^, barley and oats^68^, buckwheat^69^ and quinoa^70,71^, incentivizing functional food development using these crops in developed countries (Supplementary Table 1).

#### Minor cereals

Sorghum (*Sorghum bicolor* L.) and millets are important food items in South Asia and Sub-Saharan African countries, accounting for a large part of total caloric intake^72^. They are mainly grown in the semi-arid and sub-humid areas due to their adaptability to heat and drought conditions. Sorghum ranks fifth in the global cereal crop production followed by pearl millet (*Pennisetum glaucum*). Other millets such as finger millet (*Eleusine coracana*), kodo millet (*Paspalum scrobiculatum*), fonio (*Digitaria exilis*), proso millet (*Panicum miliaceum*), foxtail millet (*Setaria italica*), barnyard millet (*Echinochloa utilis*), little millet (*Panicum sumatrense*), and tef (*Eragrostis tef*) are also important crop species. Sorghum and millets are commonly consumed as wholegrains in traditional cuisines, such as roti (unleavened breads or pancake) and porridge. They are nutritionally analogous to conventional cereals (on average 65% carbohydrates, 10% proteins, 3.5% fat, and 8% dietary fiber) and serve as an excellent source of micronutrients (vitamins, e.g., B vitamins and vitamin E, and minerals, e.g., magnesium, phosphorous and iron), and phytochemicals (phenolic acids, tannins and flavonoids)^72^. The consumption of sorghum and millets has been linked to a multitude of health benefits, such as weight control^66^, lowering serum cholesterol and triglycerides levels^73^, reduction in starch digestibility and improvement of blood glucose control^74^, and mitigation of gastrointestinal disorders including the risk of colon cancer^75^. Sorghum and millet flours have been used at high levels, e.g., 30–60% in composite wheat bread and marketed as high fiber breads^76^, which contain an increased mineral content^77^, and a higher total phenolic content and antioxidant activity^78^. Furthermore, adding foxtail (20–50%), pearl millet (50%) or tef (40%) reduces starch digestion and absorption and therefore potentially lowers the glycemic index (GI) of composite wheat bread compared to 100% wheat bread^77,79,80^.

Barley (*Hordeum vulgare* L.) is the fourth most important cereal, and its largest producer is the European Union, followed by Russia, Ukraine, and Australia. Barley is used predominately as animal feed (ca. 70%) and to a less extent as a brewing raw material (ca. 21%), and only 6% is consumed by humans^81^. Oats are cultivated mainly in cold northern areas in Europe and North America and used primarily as livestock feed and to some extent as human food. In recent years, the consumption of barley and oat-based products (e.g., breakfast cereals, porridge, and unleavened bread) has substantially increased due to consumer awareness regarding their nutritional values (e.g., β-glucans and antioxidant compounds) and health claims. Barley and oat grains contain high levels of β-glucan, 2.5–11.3% and 2.2–7.8%, respectively^82^. The consumption of ≥3 g barley or oat β-glucan per day (i.e., 0.75 g/serving) has been acknowledged by the United States Food and Drug Administration (FDA) and the European Food and Safety Authority (EFSA) to have health claims, such as lowering postprandial glycemic and insulin responses, lowering serum cholesterol and lipid levels, immune stimulant activity, reduced risk of colon cancer, preventing type 2 diabetes, and improving gastrointestinal function (via increasing the apparent viscosity in the upper digestive tract)^83,84^. β-glucans appear to remain intact following the baking process, but high levels of oat or barley flour needs to be added to wheat dough to meet the health claims (ca. more than 50%)^85^. In a recent study, oat fiber (70% of β-glucan) was used in bread making by substituting 10–14% of wheat flour with it, resulting in a bread product with 3.4–4.6 g β-glucan/100 g serving^86^. Furthermore, the incorporation of 60% barley flour^87^ and 5–20% oat bran^88^ enriched the dietary fiber content, total phenolic content, and enhanced the antioxidant activity of the final breads.

#### Pseudocereals

Quinoa (*Chenopodium quinoa*), amaranth (*Amaranthus* sp.), and buckwheat (*Fagopyrum esculentum*) are considered the most important pseudocereals in terms of world production^5^. Quinoa and amaranth are ancient crops mainly grown in South America, such as Peru and Bolivia. Buckwheat is mainly produced and consumed in Russia and China, followed by Ukraine and the United States. These crops are climate resilient with little water demand and good tolerance against heat, drought, and soil salinity compared to cereals^89^. Furthermore, pseudocereals have a higher nutritional value than wheat and rice. Quinoa, amaranth, and buckwheat are rich in protein (on average 14%) with a well-balanced amino acid profile and are good sources of dietary fiber (14.6%), unsaturated fatty acids (4.7%), vitamins (ascorbic acid, tocopherol, carotenoids, folate, riboflavin, and thiamine), minerals, and bioactive components (e.g., polyphenols, saponins, betalains, phytosterols, and bioactive peptides)^89^. They hold potential as functional foods due to the health promoting effects that have been extensively studied in vivo and in vitro^90–93^. Some of the most consistently reported health benefits include the antioxidant, anti-inflammatory, anti-obesity, anti-diabetic, anti-cancer properties^94^; more recent studies in pre-clinical models suggest that some of the abovementioned effects are attributable to the prebiotics properties of pseudocereals, such as by promoting the growth of putatively beneficial *Faecalibacterium prausnitzii*, *Lactobacillus*, and *Bifidobacterium*^95^. Though, it remains to be seen whether the prebiotic effects of pseudocereals can withstand the baking process and be delivered to humans upon consumption.

The integration of pseudocereals into the global food production system, e.g., bread making has been promoted to offer food security owing to their current underutilization. In the study by Lin et al.^96^, buckwheat was incorporated at 15% to increase the antioxidant activity of wheat bread. Using whole amaranth flour up to 25% in baking substantially increased the protein, dietary fiber, and mineral (e.g., Fe and Zn) contents^97,98^ with relatively minor changes in the physicochemical and rheological properties of wheat dough^99^. However, a report claimed that the antioxidant activity of amaranth-wheat composite flour decreases with the increase in the percentage level (5–15%) of amaranth flour^100^. Bread enriched with quinoa flour (5–40%) had a decreased in vitro starch digestibility together with lower predicted GI than refined wheat bread^10,101^. Nevertheless, quinoa has a bitter taste due to the saponins present in the seed coats. For this reason, quinoa grains are often preprocessed to remove the saponins (e.g., by washing^101^ and dehulling^102^) before using in breadmaking to avoid the bitter flavor. It is currently unclear whether the pretreatments of quinoa affect its starch digestibility, and thereby compromising the GI-lowering benefit in the composite bread.

#### Root and tuber flours

Root and tuber crops, such as cassava (*Manihot esculenta Crantz*), sweet potatoes (*Ipomoea batatas* L.), and yams (*Dioscorea spp*.), play an important role in household (especially in rural regions) and national food security in tropical countries. The world’s total annual production of cassava and sweet potatoes during the period 2015–2020 was 291 and 91 Mt, respectively, above 60% of which was from Africa and ~30% from Asia^5^. Cassava, sweet potato, and yam flours have a high carbohydrate content (up to 90%) with considerable amounts of resistant starch. Resistant starch significantly decreased glycemic response and insulin secretion, as it is not digested and thereby acting like a sponge that slows the release of glucose from other foods^103^. Moreover, certain types of resistant starch are well-established prebiotics with consistent effects across individuals^104^. The use of cassava flour in wheat bread making has been promoted by Nigeria and Brazil, the world leading producers of cassava roots, to reduce wheat importation^36^. Depending on the type of cassava flour, up to a 30% substitution level has been used to produce acceptable cassava-wheat composite bread^105,106^. However, the addition of fibers from an external source is needed to meet the recommended daily intake, as cassava has a very limited content of fibers^106^. Sweet potato flours (particularly the orange-fleshed types) are rich in β-carotene, the most important provitamin A carotenoid, and have been exploited as a promising strategy for preventing vitamin A deficiency in developing countries^107^. In bread preparation, the replacement of wheat flour by 10–30% orange-fleshed sweet potato flour increased the dietary vitamin A content^108,109^. Consuming 100 g of the composite bread with 30% orange-fleshed sweet potato flour would meet 89% of the recommended dietary allowance (RDA) of vitamin A for children aged 3–10 years and 50% of the RDA for pregnant women^110^. Yam flour has shown potential in reducing the GI value of breads. For example, the inclusion of yam flour (purple types) at 30–50% increased the resistant starch content of wheat bread and decreased the in vitro starch digestibility^111^. Yam flour is also rich in bioactive compounds, e.g., mucilage and phenolic compounds, which have been utilized to increase the antioxidant properties of composite bread^112^.

### Waste valorization: surplus bread and brewers’ spent grain

The FAO reported that around one-third of food produced for human consumption every year is lost or wasted globally, which has become a challenge for economic development in both industrialized and developing countries. Bread, susceptible to staling and microbial spoilage, is among the most wasted food groups due to its large production volume and short shelf life. Surplus bread refers to bread that is manufactured, retailed or served but is not sold to or consumed by customers. In Finland, roughly 5–10% (10–20 million kg) of the bread products produced annually are wasted before entering retail and reaching consumers^113^. In the United Kingdom, bread waste accounts for 11% of all food waste generated as estimated by the Waste and Resources Action Programme (WRAP), the majority of which comes from the household waste i.e., 20 million bread slices per day^114^. In Sweden, annual bread waste amounts to ~80410 tonnes, i.e., 8.1 kg per capita per year^115^. Common waste treatment options for surplus bread include bioethanol, animal feed, and beer production, anaerobic digestion and composting, landfill (methane), and incineration^115^. We recently reported a promising alternative where the edible bread waste was recycled and transformed into fresh baked products^113,116^. The recovery of bread waste for human consumption meets the concept *waste* = *food* and is a sustainable solution to minimize food waste and its environmental impact, contributing to the United Nations Sustainable Development Goal (SDG) 12.3, i.e., 50% reduction of the worldwide food waste at the retail and consumer levels and reduction of food loss along production and supply chains by 2030.

Brewer’s spent grain (BSG) is the principal byproduct obtained from the beer-brewing process with an estimated annual production volume of 39 Mt globally^117^. The conversion of barley grains into beer can be divided into 3 phases: malting, mashing, and yeast fermentation. In the mashing stage, the barley malt is hydrolyzed by enzymes to produce the sugar-rich liquid (known as wort) that will be further fermented into beer. The insoluble unhydrolyzed part, also known as solid BSG, is collected after the lautering step in the mash tun. BSG is composed of mainly barley husks, pericarp, and seed coat fractions. The nutrient composition of BSG depends on the barley variety and the malting and mashing conditions^118^. In general, BSG contains 14–31% (dry matter) protein, 0–12% starch, 0–13% fat, and a substantial amount of hemicellulose (up to 42%, mainly arabinoxylan), cellulose (33%), and lignin (11–22%)^118^. The essential amino acids account for ca. 30% of the total protein content of BSG with lysine being the most abundant^117^. Minerals (e.g., silicon, phosphorus, and calcium) and vitamins are also copiously present in BSG. Currently, BSG is mainly used as animal feed. However, its high nutritive value, low cost, and availability have prompted recent research aiming to incorporate BSG into human food. The consumption of BSG has been suggested to confer health benefits (e.g., lowering postprandial glycemic responses and cholesterol levels), potentially attributable to the fibers arabinoxylan and β-glucans and phenolic components^118^. A recent study using in vitro fermentation suggests the prebiotic potential of BSG^119^. BSG have been milled into flour as a protein-rich and fiber-rich ingredient in wheat bread making at levels up to 20%^11,120–122^, which holds potential as a sustainable strategy for waste management.

## Techno-functional, sensory and nutritional challenges in bread making using wheat flour substitutes

### Key features in wheat bread making

The textural and sensory qualities of wheat bread are often considered as a benchmark for composite bread. Wheat bread making is a multistage dynamic process with several essential features, including mixing of the ingredients, development of a gluten network from kneading, incorporation of air bubbles, fermentation in which CO_2_ produced by yeast is entrapped in air bubbles, baking, crust formation, surface browning reaction, and formation of the cellular structure in final bread^1^. Upon mixing, the gluten proteins (i.e., gliadins and glutenins) are hydrated and a three-dimensional gluten network (disulfide (SS) bonds) is formed with air cells being trapped in this matrix. During yeast fermentation, the produced CO_2_ dissolves in the aqueous phase of the dough until saturated, and then diffuses to the existing cell nuclei while some CO_2_ escapes. The retention of gas bubbles is essential for the liquid foam structure of the dough. The gluten network, which creates the viscoelastic properties of bread dough, plays a crucial role in gas holding and dough development^1^.

### Techno-functional challenges in composite bread making

Flours of other crops may not be conventionally processed in bread making due to significantly different properties of their proteins compared to wheat gluten. Using wheat flour substitutes in bread making at high levels usually produces final products of unacceptable quality. In general, the substitution levels above 10% lead to a decrease in bread specific volume and an increase in crumb hardness (Supplementary Table 1). The effects of adding non-wheat flours on the rheological properties of dough, e.g., farinograph water absorption, starch pasting profiles, dough extensibility, and viscoelasticity (elastic modulus and viscous modulus), have been extensively investigated. The addition of fiber-rich ingredients derived from legumes, barley, oats, and BSG often results in increased water absorption, whereas the opposite effect occurs with the addition of starchy ingredients, such as millets and root flours. Moreover, incorporating wheat flour substitutes at high levels leads to longer dough development time, higher starch gelatinization temperature, lower dough stability and extensibility, decreased gluten strength and elasticity, and increased dough stickiness^45,47,62,80,112,123–127^. These negative impacts are related to a weakened gluten network, where (1) gluten protein hydration is reduced due to the competition of water between gluten proteins and fibers or non-wheat proteins; (2) the formation of the gluten network is disrupted due to the different functional properties of non-wheat proteins; (3) the gluten secondary structure is altered^46,80,128^.

### Sensory challenges in composite bread making

Consumers crave foods that satisfy the sensory qualities they enjoy, such as mouthfeel, taste and aroma. Flavor is the combination of aroma, taste and chemesthesis. Taste is due to the non-volatile compounds present in food described as sweet, salty, bitter, sour, and umami. Aroma is related to volatile compounds. The chemesthesic sensations are usually perceived through the stimulation of the human trigeminal nerve endings within the mouth, nose, or eyes^129^. The off-flavors present in wheat flour substitutes, such as beany flavor, bitter taste, and aftertaste represent a major hindrance toward consumer acceptability^130^. The enrichment of wheat bread with legume-based ingredients at higher levels often leads to a beany flavor. For example, the inclusion of soy flour above 10% generated a strong beany flavor and an aftertaste, resulting in lower flavor ratings and taste acceptance than the wheat control^131^. A decrease in sensory scores for odor, taste, and overall acceptability was reported when more than 10% of chickpea flour or red kidney bean flour was included in wheat bread^123,132^. The incorporation of 10% lupin protein isolate generated beany, earthy, and malty notes in the bread^133^. Legume seeds contain 2–20% lipids with a high level of unsaturated fatty acids: oleic (4–38%), linoleic (28–55%) and linolenic (3–37%) acids^134^. The oxidation of unsaturated fatty acids plays a crucial role in the development of off-flavor compounds in legume-based products^129^. This oxidation can be enzymatic or non-enzymatic (auto-oxidation and photo-oxidation). Legumes, e.g., soy, faba bean, and pea, are rich sources of lipid degrading enzymes, such as lipoxygenase and lipase. Lipase catalyzes the hydrolysis of triglycerides to free fatty acids. Lipoxygenase catalyzes the degradation of polyunsaturated fatty acids to produce hydroperoxides, which are subsequently degraded in enzymatic or chemical reactions forming volatile and non-volatile compounds responsible for off-flavors^135^. Hexanal, 3-*cis*-hexenal, 2-pentylfuran, (*E*,*E*)-2,4-decadienal, and ethyl vinyl ketone are identified as major lipoxygenase-derived contributors to beany and green notes^135^. These off-flavor compounds are detected at low threshold values and thus a small quantity of fatty acids is enough to develop a strong beany off-flavor.

Bread enriched with wholegrain or fiber-rich ingredients has the organoleptic characteristics often described as bitter, astringent, and rancid, which is related to the presence of free fatty acids, saponins, alkaloids, isoflavones, phenolic acids, tannins, small peptides, or amino acids, or combinations thereof^129^. Sorghum contains a significant amount of polyphenols and condensed tannins contributing to bitterness and astringency^136^. The addition of 50% wholegrain sorghum flour in wheat bread led to higher intensities of bitter taste and aftertaste compared to 100% wheat bread^137^. Oat flour, having a high lipid content (4–8%), is susceptible to lipid oxidation where the produced long-chain hydroxyl fatty acids confer a bitter taste, and its volatile compounds impart a rancid off-flavor^138^. The incorporation of 5–15% barley protein isolate induced an intense bitter taste of wheat bread^139^. BSG has a typical malt flavor developed during the mashing process and a bitter taste^140^. Bread supplemented with BSG at above 10% had more intense bitterness and acidic flavor^11^. Adding BSG or oat bran to wheat flour at levels higher than 10% reduced the sensory scores for odor, taste and overall acceptability^88^. Legumes, such as faba bean, lentil, and soy, contain a considerable amount of saponins (saponin βg and saponin Bb), which are perceived as bitter, astringent, and metallic^135^. Lupin (*Lupinus albus* L.) is rich in alkaloids with a strong bitter taste and needs to be debittered prior to bread making^50^. For this reason, the Australian sweet lupin (*Lupinus angustifolius*), which contains very low levels of bitter alkaloids, is a preferred option in bread fortification^141^. Furthermore, the off-taste compounds and precursors in plant raw materials are often retained during the protein isolation process due to their interactions with proteins (e.g., bitter-tasting kaempferol derivatives in rapeseed protein isolates), causing a negative sensory perception^142^.

### Nutritional challenges in composite bread making

Plant-based ingredients contain certain phytochemicals naturally produced as secondary metabolites by plants^143^. As part of the plants’ defense mechanism against being eaten, these bioactive compounds almost always confer off-tastes in addition to disrupting the bioavailability and utilization of nutrients and minerals in animals^143^, and hence are dubbed as “antinutrients”. Antinutrients are sometimes referred to as non-nutrients since some studies claim that they possess health promoting effects when in the appropriate quantity and under the right conditions^144^. Notwithstanding their ambivalent properties that require further research, elimination or reduction of antinutritional factors is the target in most food production. Oilcakes contain antinutrients such as phytic acid, glucosinolates, sinapine, cyanogenic glycosides, trypsin inhibitors, and tannins^145^. Sinapine (bitter taste) is the major phenolic constituent in rapeseed meals, which forms complexes with proteins via oxidation and decreases digestibility^145^. Glucosinolates (bitter taste) have been shown to have goitrogenic and anti-thyroid effects in both humans and animals^144,145^. Cyanogenic glycosides (bitter taste), the principal antinutrient in flaxseed meals, can produce toxic hydrogen cyanide following the breakdown in the gastrointestinal tract^146^. Linatine is also found in flaxseed meals that can cause pyridoxine (vitamin B6) deficiency^146^. Phytic acid, present in most oilcakes, can bind to minerals, proteins, and amino acids. This reduces their bioavailability and inhibits the activity of α-amylase, leading to decreased starch digestibility. Tannins (bitter and astringent) can precipitate proteins and reduce the absorption of minerals, particularly iron. Trypsin inhibitors are known to reduce the digestibility of proteins^145^. Legumes also contain high concentrations of antinutrients such as phytic acid, lectins, vicine and convicine, enzyme inhibitors (trypsin, chymotrypsin, and α-amylase inhibitors), condensed tannins, saponins, and flatulent-causing oligosaccharides^147^. Lectins are carbohydrate-binding proteins widely distributed in leguminous crops. Legume lectins negatively affect the functions of human digestion system and nutrient absorption due to their binding to the intestinal epithelial cells^147^. Vicine and convicine cause a severe haemolytic anemia, known as favism, in susceptible individuals with the deficiency in the glucose-6-phosphate dehydrogenase enzyme^148^. The indigestible raffinose family oligosaccharides (RFOs), such as raffinose, stachyose, and verbascose, are abundant in legumes. While several studies suggest their prebiotic potential, the high intake of RFOs causes abdominal discomfort and diarrhea in some people via gas production derived from increased colonic fermentation^149^. Pseudocereals contain saponins, phytic acid, tannins, and protease inhibitors^150^. Saponins are particularly abundant in quinoa, which cause hemolysis by reacting with the sterols of erythrocyte membrane and interfere with the absorption of lipids, cholesterol, bile acids and fat-soluble vitamins^151^. Phytic acid and tannins are major anti-nutritional components present in sorghum, millets, and BSG^152^.

## Strategies to improve textural, sensory and nutritional attributes of composite bread

**Fig. 4:**
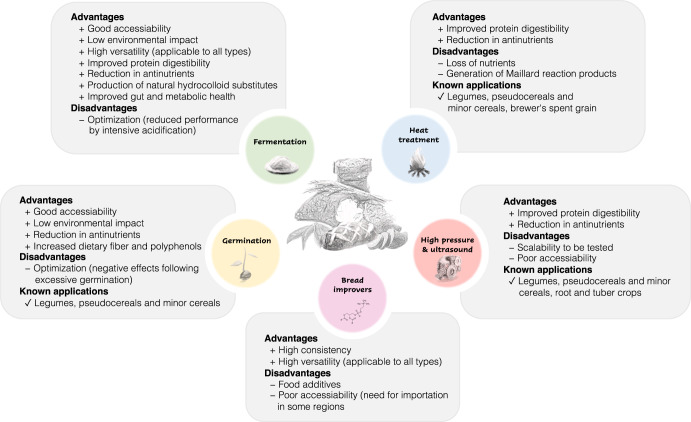
Summary of important processing strategies for improving sensory and nutritional quality of composite bread.

The altered techno-functionality, sensory characteristics and presence of antinutrients in wheat flour substitutes represent a major limitation in their utilization and eventual consumer acceptability. Several processing strategies have been applied to produce composite bread with technological and sensory profiles comparable to refined wheat bread. Main advantages and drawbacks of the different processing strategies, in addition to textural and sensory improvements, are summarized in Fig. 4. Few strategies are universally effective for all types of wheat flour substitutes, and therefore optimization of conditions for specific ingredients or combinations of strategies are often needed to achieve desirable outcomes.

### Formulation-based approaches

#### Bread improvers

The addition of wheat gluten or bread improvers that mimic the viscoelastic and gas retention properties of gluten, such as hydrocolloids, emulsifiers, and enzymes, is the most straightforward and widely used method in composite bread making. For example, the composite bread containing 61.8% barnyard millet, 31.4% wheat, and 6.8% gluten exhibited comparable textural and sensory attributes to refined wheat bread^153^. Hydrocolloids, such as hydroxypropylmethylcellulose (HPMC), carboxymethylcellulose (CMC), guar gum, gum arabic, xanthan gum, and pectin, have been extensively used in composite bread formulations to modify dough rheology and improve bread volume and crumb softness^130^. The functional properties of hydrocolloids are contingent on their high water-binding capacity and interactions with dough structural components, e.g., gluten proteins and starch. Emulsifiers, such as sodium stearoyl-2-lactylate (SSL), diacetyl tartaric acid esters of monodiglycerides (DATEM), and lecithin (LC), function as dough strengtheners, texture enhancers, and anti-staling agents in composite bread^154,155^. Various enzymes with hydrolytic or cross-linking functions, such as amylases, xylanases, cellulase, and transglutaminases, are often employed alone or in conjunction with other improvers to achieve better textural properties^126,155^. Amylases are an important class of enzymes used to increase gas production by generating fermentable sugars for yeast, thereby producing higher-volume loaves. Amylases can also retard bread staling by preventing amylopectin retrogradation. For example, the hydrolysis of gelatinized starch in surplus bread using α-amylase and amyloglucosidase improved the baking performance and bread quality^113^. Xylanases degrade water-unextractable arabinoxylans into soluble forms and thus improve loaf volume and softness. Transglutaminases enhance the formation of protein networks through the formation of crosslinks^155^.

While these bread improvers have proven consistent effects and are applicable to different wheat flour alternatives, most of them are classified as food additives that are perceived with a negative connotation. Importantly, a growing body of evidence suggests that the use of some improvers has previously unknown ramifications in health and disease. For example, a recent randomized controlled trial in healthy adults showed that a diet enriched with the emulsifier CMC reshaped the intestinal milieu with negative changes in the gut microbiota^156^. Xanthan gum, an effective thickening agent, emulsifier and stabilizer commonly used in gluten-free bread, has been shown to select for the xanthan-degrading capacity in the gut microbiota found only in industrialized populations but not in hunter-gatherers, the health implications of which are unclear but highly relevant for people who regularly consume above-average amounts of added xanthan gum, e.g., patients with celiac disease^157^. Other drawbacks associated with bread improvers include the need and high cost for importing commercial bread improvers in developing countries^36^, and that the addition of improvers does not reduce the antinutrients originating from the plant sources.

A few clean-label (E-code free) and accessible alternatives to bread improvers have been proposed. One approach involves adding locally available natural ingredients (e.g., fruits) rich in ascorbic acid that acts as an oxidizing agent in strengthening the gluten^158,159^. For instance, a Kuwaiti study by Zafar et al. demonstrated that the addition of *amla* (*Phyllanthus emblica*; widely grown in South Asia and Middle East) powder improved the mixing characteristics, specific loaf volume, sensory qualities and overall acceptability of wheat–chickpea composite bread^158^. Lactic acid fermentation by particular bacterial strains can produce natural hydrocolloid substitutes, e.g., dextran, that improve the techno-functionality of composite dough, which will be discussed in the next section.

### Technological approaches

#### Heating treatment

Heat treatments (dry and wet), such as drying, roasting/toasting, steaming and extrusion cooking, can reduce the antinutritional compounds, e.g., tannins and phytic acid in plant-based materials^144,148^. Thermal denaturation leads to the inactivation of protease inhibitors and lectins, exposing the susceptible sites of proteins to proteolysis and thereby improving protein digestibility^160^. Thermal treatments may also inactivate the lipid modifying enzymes and therefore prevent lipid oxidation. Among the thermal processing methods, roasting represents an economical technique that can be performed on a large scale. Roasting is particularly effective in eliminating the unpleasant odorants and generating the desirable aromas, e.g., pyrazines and alkylated pyrazines in legume-based ingredients. The composite bread using roasted yellow pea flour (10–20%) exhibited a pleasant roasted-like flavor with less beany, earthy, and grass-like off-flavors compared to its control^45,161^. Roasting reduced the levels of oligosaccharides and increased protein digestibility of chickpea, pea, lentil, and soy (30%), leading to composite bread with more acceptable aromas and a higher specific volume^160^. The addition of roasted pea flour (30%) also improved the volume and texture of composite bread to levels comparable to refined wheat control^162^.

Low-moisture extrusion cooking is a high-temperature (e.g., 110–160 °C) short-time process, which has been used to modify flour properties through starch gelatinization, protein denaturation or aggregation, interactions between amylose and lipids, and solubilization of dietary fiber^163^. Extrusion increased starch digestibility due to gelatinization and altered starch structure, which may have implications in the glycemic index^163^. During extrusion cooking, the feed moisture and die temperature are important parameters affecting the functional properties of flour extrudate. The extruded flours as pre-gelatinized starch can be used as an alternative to food hydrocolloids or gluten in bread products. For instance, the extruded mung bean (20%)^164^, sorghum (10%)^165^, finger millet (20%)^166^, and BSG (12%)^126^ resulted in composite doughs with higher water absorption and viscoelastic properties, and thereby improved bread textural quality and sensory acceptability. Other heat treatments in the presence of moisture, such as by tempering non-wheat grains followed by incubation at 20–70 °C for different durations (up to 10 days), have been used to achieve better textural and nutritional qualities of millet-wheat composite bread^78^. On the other hand, thermal treatments inevitably cause the loss of heat-labile nutrients, such as vitamins^167^. Significant reduction of heat sensitive phenolic acids and flavonoids has been reported in heat-treated fours made of various non-wheat crops, nullifying their high antioxidant activity originally present in untreated flours^168^. A recent study by Ciesarová et al. demonstrated that thermally treated wheat flour substitutes, especially by roasting, contributed to higher contents of potential carcinogens acrylamide and 5-hydroxymethylfurfural in final breads^169^. These Maillard reaction products (MRPs) also have been associated with negative metabolic health^170^. Future studies are warranted to develop sufficiently effective thermal treatments for improving the breadmaking potential of non-wheat flours, while minimizing the formation of certain harmful MRPs.

#### Germination

Germination under controlled conditions is an economical, accessible and environmentally friendly approach to improve nutritive and techno-functional properties of wheat flour substitutes made from legumes and cereal grains. During germination, the endogenous enzymes, e.g., proteases and amylases are activated that hydrolyze proteins, starch, and lipids, leading to improved digestibility and bioavailability of essential nutrients^171^. Studies have shown that germination can increase protein and total dietary fiber, reduce tannin and phytic acid contents and increase mineral bioavailability in legumes^172,173^. Plant seeds such as legumes and buckwheat are able to accumulate selenium (Se) and convert it to its organic form, e.g., selenomethionine. Biofortification of Se may be achieved by soaking the legume seeds in Na_2_SeO_3_ contained medium during the germination process, which is also a promising way to increase the antioxidant activity of the resultant composite bread^174^. The use of germinated flours in wheat bread formulations leads to changes in dough rheological properties and bread quality. In general, the addition of germinated flours at levels higher than 10% negatively affects bread volume and textural attributes, whereas beneficial effects have been shown below the 10% addition level^174,175^. Germination has a profound impact on the flavor profiles of the legume seeds and their bread products, including a stronger sweet taste due to increased sugar concentrations and green, mushroom, and meaty odors due to the release of particular volatile compounds^129^. Germinated barley flour (24 h) added at 30% improved the flavor and textural attributes and overall acceptability of the composite bread compared to its counterpart made of untreated barley flour^176^. However, excessively germinated barley (≥48 h) was shown to unfavorably affect the gluten network development and textural formation of bread, though the total phenolic content and antioxidant capacity of the bread were significantly elevated^176^. Excessively germinated legumes were described as intensive beany flavor due to the higher lipoxygenase activity and bitter taste due to the release of polyphenols^177,178^.

#### Fermentation

Fermented foods have long been an integral part of the human diet in nearly every culture. Sourdough, a leavening agent obtained from a mixture of flour and water and spontaneously fermented by a complex microbiota dominated by various strains of lactic acid bacteria (LAB), acetic acid bacteria (AAB) and yeasts, was an important component of traditional bread making until the widespread use of industrially produced yeast and chemical yeast agents. Fermentation has gained renewed interest for manufacturing high-quality composite bread due to the technological and nutritional benefits and the advantage of being accessible by bakers from low- and middle-income countries and widely accepted by global consumers on the basis of familiarity.

Refined wheat or composite bread made by sourdough fermentation has consistently been shown to attenuate the GI across multiple cohorts, in both healthy individuals and adults with impaired glucose tolerance, potentially due to multiple mechanisms, e.g., increased resistant starch and synthesis of free phenolic compounds^179^. Recent in vitro studies suggest that the consumption of bread made with the lactic acid fermentation process promotes the production of gut microbial metabolites, short-chain fatty acids, which are putatively beneficial for metabolic health^180^. The incorporation of fermented flour also improves the nutritional quality of the composite bread with less antinutritional factors, e.g., phytic acid, condensed tannins, and oligosaccharides. These positive effects partly emanate from the metabolic activities of LAB and the activation of plant-derived enzymes or microbial enzymes, which results in organic acid production, proteolysis, and the formation of antimicrobial compounds. For example, the addition of 20–30% chickpea protein fermented with *Pediococcus acidilactici* exhibited a 75–90% reduction in raffinose, stachyose, and verbascose compared to the unfermented composite bread^181^. Wheat bread formulated with 15% (flour weight) fermented legume flours from chickpea, lentil and bean and 30% fermented faba bean flour showed higher in vitro protein digestibility, free amino acids, and a lower predicted glycemic index^44^. Buckwheat, amaranth, chickpea, and quinoa flours fermented with *Lactobacillus plantarum* C48 were used to produce a functional bread enriched with γ-aminobutyric acid (GABA, the major inhibitory neurotransmitter of the central nervous system)^182^. The incorporation of 20–50% fermented foxtail millet flour into bread formulations produced low-GI composite bread (GI < 55)^79^. The use of 12.5% quinoa flour fermented with *L. plantarum* T6B10 and *L. rossiae* T0A16 improved the total dietary fiber content and sensory properties of composite bread^183^. Fermentation also reduced the degree of β-glucan depolymerization in oat-wheat composite bread by lowering the activity of β-glucan-degrading enzymes^184^.

**Fig. 5:**
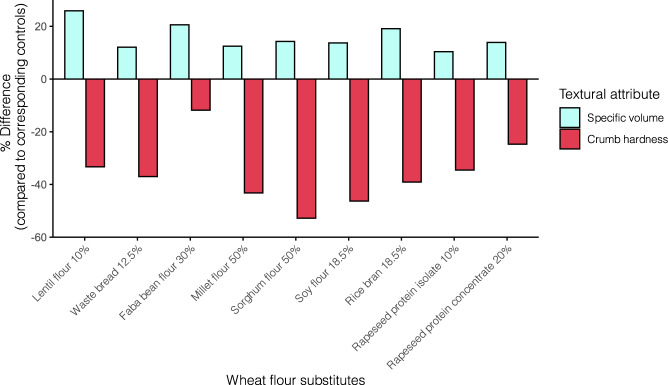
Improvement (expressed as % difference) in specific volume and texture of various types of composite bread by fermentation with dextran-producing *Weissella confusa* strains.

The capability of microbes in producing a myriad of metabolites can be capitalized by fermentation tailored to improve the textural quality of bread. The texture-enhancing dextrans are high molecular weight natural polymers, synthesized by dextransucrase enzymes from LAB using sucrose as the substrate^185^. Dextrans produced by the dextransucrase from the *Weissella* strains have a linear structure with ~97% α-(1 → 6) linked D-glucosyl units and 3% α-(1 → 3) branch linkages^130^. These dextrans are natural alternatives to commercial hydrocolloids that have been previously reviewed by us regarding how they positively influence rheological and textural properties of composite dough, for instance, by increasing water absorption and dough viscoelastic properties^130^. Furthermore, fermentation with the *Weissella* strains leads to mild acidification, which favors the functionalities of in situ-produced dextran^130^. Recent work has demonstrated that in situ-produced dextran derived from fermentation represents a sustainable approach to improving the textural quality of composite bread made of a variety of wheat flour substitutes^46,80,113,131,137,186,187^ (Fig. 5).

Fermentation reduces the beany flavor and bitter taste of legume-based ingredient via degradation of bitter-tasting compounds, e.g., saponins and alkaloids, decreased lipoxygenase activity at a low pH, and conversion of aldehydes or alcohols into the corresponding acids by the aldehyde dehydrogenase or alcohol dehydrogenase from LAB^129^. Our recent studies explored the off-flavor masking effects of in situ-produced dextran in sorghum, soy flour, rice bran-enriched wheat bread^131,137^ as well as in the composite bread made of rapeseed protein^65^. The flavor-modifying effects by dextran are likely owing to the binding of flavor compounds to dextran, and/or the altered oral texture of the food products, e.g., enhanced cohesiveness, entrapping the flavor molecules within the food matrix during chewing^131,137^. The flavor-masking effects of dextran are concentration dependent, i.e., the taste and aroma intensities are reduced when dextran is added at levels higher than the critical coil overlap concentration (c*). Furthermore, the positive influence of dextran on bread’s flavor and texture can be counteracted by intensive acidification, e.g., with a long fermentation time^188^ or the use of *L. pseudomesenteroides* as starters^46^. Intense sourness in food products is generally unpalatable. Therefore, when applied to novel ingredients, optimization of fermentation conditions based on ingredients’ specific characteristics is required to achieve mild acidity with a sufficient level of in situ produced dextran.

Importantly, the versatility of fermentation allows the integration of other processing methods designed for specific purposes or for wheat flour substitutes with hardly managed technological properties. For example, in our recent work, a mixed-fermentation approach was applied to soya flour or rice bran using *Propionibacterium freudenreichii* DSM 20271 and *Weissella confusa* A16 to produce composite bread of high sensory attributes with adequate active B12 produced by *P. freudenreichii* that meet the recommended daily requirement^131^. As plant-based foods are generally deficient in B12, strategies of this kind hold great potential for combating the “hidden hunger” associated with wide-scale micronutrient deficiencies. On the other hand, composite bread with better nutritional and sensory properties may be accomplished by the combination of fermentation with other processing methods, e.g., with germination for legumes, underutilized cereal and millet grains^189–191^ or with debittering for lupin^192^.

#### Other emerging technologies

In recent years, innovative processing technologies, such as high hydrostatic pressure treatment (i.e., high pressure processing) and ultrasound treatment, have been repurposed for valorizing non-wheat ingredients in bread making. High hydrostatic pressure treatment is a non-thermal pasteurization technology used to destroy microorganisms and inactivate enzymes in foods. Food products are subjected to hydrostatic pressures (100–1000 Mpa) for a short period using water as the pressure transfer medium^59^. High pressure processing modifies properties of starch gelatinization, denatures and aggregates protein, resulting in enhanced strength and viscoelastic properties of composite dough^193^. High pressure processing also improves starch and protein digestibility and reduces antinutritional factors of legume- and cereal-based products. High pressure processing (350 MPa, 10 min) has been applied to hydrated cereal flours e.g., oats, millets, and sorghum (40–60%) to improve sensory qualities, the antioxidant potential, and starch digestibility of the composite bread products^193^.

The ultrasound technique has been claimed to be a vital technique to help achieve the aim of sustainable “green” chemistry. This non-thermal technique operates on the principle of acoustic cavitation generated by high-intensity and low-frequency sound waves (20–100 kHz), and can be used in a wide range of applications, including fermentation, emulsification and extraction^59^. The ultrasound treatment modifies protein solubility and functionality, inactivates endogenous microbes and enzymes in flour, and assists the extraction of phenolic compounds and alkaloids from legumes, i.e., debittering by damaging the plant matrix cell walls. The use of ultrasound treated flour in composite bread making is relatively nascent. In the study by Yaver and Bilgiçli, the use of lupin flour (10–20%) debittered by ultrasound increased the specific volume and decreased crumb hardness of the final composite bread compared to the flour treated by the traditional debittering method^50^. Sweet potato pulp-wheat composite dough (50:50) treated by ultrasound displayed improved rheological properties, which produced a bread product with a higher bread specific volume and softer crumb compared to the untreated controls^194^. While the abovementioned processing technologies are environmentally friendly and supposedly efficacious, most studies were performed in the laboratory settings. Therefore, their scalability and accessibility, especially in low- and middle-income countries, remain to be tested.

## Future prospects

Thus far, we have discussed the benefits and challenges associated with bread making using various types of wheat flour substitutes, the demand and research of which will likely increase exponentially in the near future. Going forward, interdisciplinary approaches addressing the current knowledge gaps in the environmental, nutritional, health and technological dimensions are required. The synergism between sustainability science, human nutrition, microbiomics and food science is necessary to scale up research results for large-scale positive impact^195^.

In the environmental dimension, life cycle assessment (LCA) studies on the ingredient-to-bread chain are warranted to understand the environmental impact of composite bread made of different wheat flour substitutes. A comparative approach simultaneously evaluating the nutrient density per unit environmental impact per serving should be devised to identify candidates that are both nutritionally dense and environmentally sustainable. Chaudhary et al. developed the nutrition carbon footprint score (NCFS) as an indicator of product-level nutrient density per unit environmental impact by combing nutritional profiling systems with LCA analysis^13^. Such nutritional profiling systems can be flexibly adapted to the nutritional needs of specific regions. For instance, in the Chaudhary study, the nutrient balance concept (NBC) was used, in which an aggregated measure is calculated based on nutritional quality, i.e., whether a nutrient is considered to have a positive or negative effect on the nutritional profile of a given food^196^. Using this approach, the authors proved that the food products made of yellow pea-wheat composite flour had higher nutrient density per unit environmental impact compared to their refined wheat counterparts^13^. The LCA analysis incorporating nutrient density is particularly important for comprehensively understanding the benefits of food waste valorization, as processing food waste into edible ingredients may increase its environmental impact in some scenarios^197^. Future studies of this kind are essential as basis for policy making involving different stakeholders to improve the knowledge of *what to eat* and develop relevant processing technologies for sustainable food production that promotes wellness, especially in vulnerable regions.

Dietary transitions toward greater consumption of healthier foods would generally improve environmental sustainability^37^. Moreover, nutritional and health benefits are instrumental in promoting the acceptance of sustainable composite bread. Consumers from developed countries are increasingly interested in selecting “gut-friendly” bread on the market^198^. These underscore the importance of conventional food trials to evaluate the effect of food products, e.g., newly developed composite bread, on consumer health^199^. Traditionally, nutritional studies have taken a reductionist approach, focusing on the constituent nutrients of a food; food science and technology has been based on a whole-food approach, placing a greater emphasis on food morphometry and physico-chemical properties. The multiplicity of interactions between nutrients in whole foods often change their nutritional performance and health potential^200^. Therefore, it has been proposed that future research needs to unite the two approaches, using “food” as a fundamental unit to investigate its effects on multiple surrogate endpoints^201^, including the gut microbiota. Albeit with inter-individual variation, a person’s gut microbiota can be used to gauge their health status^202^, predict their short-term and long-term metabolic response to diet^203,204^, and potentially foretell the risk of cardiometabolic diseases^205,206^. Therefore, it is necessary to include microbiomics in all conventional food trials, as it broadly reflects health consequences of food products and their processing technologies. The latter has not been rigorously evaluated^201^, while the relationship between processing and the food matrix, and the resulting implications in digestion, nutrition and health are a subject of recent interest. For instance, multiple studies have shown that processing techniques in bread making have a significant impact on post-prandial metabolic responses^202^. Currently, studies on the effectiveness of different processing techniques in reducing antinutritional compounds and their health implications in composite bread are lacking.

In terms of processing technologies, strategies to modify processing variables during breadmaking, such as lactic acid fermentation, remain underutilized for bread preparation from non-wheat grains^9^. We believe that fermentation with in situ produced dextran is one of the most versatile and accessible approaches for improving textural and sensory properties of composite bread. Thus, future studies would benefit from a mix-and-match approach, where the investigations focus on what optimal combination between fermentation and other methods is required to increase the proportion of wheat flour substitutes with minimum impact on the nutritional and sensory attributes of the bread. Fermentation of plant-based ingredients, either by autochthonous microbes present in the raw material or with selected starters, has been traditionally used in preparing foods and consumed in many indigenous communities in Africa, Asia, Europe, and the Americas^207^. The cultural resurgence of sourdough provides an excellent example, showcasing that these traditional fermented foods represent a treasure trove of resources that could be harnessed to improve health and food quality. A recent study profiling the microbiotas in a large collection of sourdough starters found that acetic acid bacteria, a mostly overlooked group of sourdough microbes, are responsible for the variation in dough rise rates and aromas^208^. It is therefore tempting to characterize the microbial communities in different fermented foods and identify specific microbes responsible for their unique flavors and aromas. The fermentation process can be subsequently adjusted to produce bread products with organoleptic properties similar to the fermented foods with which locals are familiar. The familiarity will likely increase local consumers’ preference even if the bread products are subpar to refined wheat bread in some aspects, as demonstrated by the previous study on bambara–wheat composite bread^51^.

Bread was first made of locally available wild wheat, wild barley and root plants before the Neolithic Revolution^209^, but has since evolved to rely on one single crop that occupies the most arable land on the planet. As a dependable staple food, this has contributed to biodiversity loss and lower environmental productivity with irreversible damage to planetary health. Transition to sustainable bread production requires multifaceted approaches, including the use of wheat flour substitutes from sustainable sources, which may be bolstered by evidence-based knowledge and translation of current and future advances outlined in this review.

## Supplementary information

Supplementary Table 1

## Data Availability

Data sharing not applicable. This is a review article and no new datasets were generated or analyzed during this study.
