# Supplementary material for: Sustainable plant-based ingredients as wheat flour substitutes in bread making
Source: NPJ Sci Food. 2022 Oct 28;6:49. doi: 10.1038/s41538-022-00163-1 (PMC9614748; doi:10.1038/s41538-022-00163-1)
Supplement: Supplementary file 1 — Supplementary Table 1 [file 41538_2022_163_MOESM1_ESM.pdf]

Supplementary Table 1 Summary of wheat flour substitutes and their influences on dough rheological and bread properties

Source material	Formulation type	Replacement level (%flour weight)	Influences on dough properties	Influences on sensory and nutritional attributes of bread	References
Legume-based					
Chickpea	Flour	5−30%	-Increased water absorption and dough development time.<br>-Decreased dough extensibility and resistance to deformation.<br>-Above 10% replacement leading to sticky dough, higher onset and peak gelatinization temperatures, and higher elastic and loss moduli.	-Increased protein digestibility and improved amino acid profiles compared to wheat control.<br>-Above 10% substitution leading to reduced specific volume and increased hardness, higher densities, and darker crumb.<br>-Textural quality of chickpea-wheat bread improved by adding 1% emulsifier.	Cappelli et al. 2020; Guardado-Félix et al. 2020; Kotsiou et al. 2022; Mohammed et al. 2012; Yamsaengsung et al. 2010
Chickpea	Protein concentrate	20−30%	-Increased dough mixing time.	-Decreased bread specific volume and denser crumb structure.<br>-Fermentation leading to reduced raffinose, stachyose, and verbascose contents by 75.4%, 97.6%, and 90.0%.	Xing et al. 2021
Lentil	Flour	5−24%	-Reduced dough tenacity, extensibility and strength.	-Increased contents of lysine-rich proteins, dietary fiber, and phenolic compounds.<br>-Enhanced antioxidant potential compared to wheat control.<br>-Above 10% substitution reducing specific volume with increased density.<br>-Aroma profile and specific volume improved and crumb hardness decreased by fermentation with in situ dextran production.	Gallo et al. 2022; Perri et al. 2021; Turfani et al. 2017
Faba bean	Flour	25−40%	Reduced dough consistency, gluten strength value, resistance to extension and dough strength.	-Increased contents of proteins, minerals, ash, total phenolic compounds, condensed tannins, and antioxidant potential.	Coda et al. 2017; Benayad et al. 2021; Wang et al. 2018

				-Protein digestibility and free amino acid profile increased, and predicted glycemic index decreased by fermentation.	
Lupin	Flour	3−20%		-Specific volume and crumb softness increased by fermentation with in situ dextran production.<br>-Increased protein, dietary fiber, phenolic, and carotenoid contents, and higher antioxidant potential.<br>-Increased protein digestibility and improved amino acid profile.<br>-Decreased in vitro glycemic index.<br>-Above 10% addition decreasing specific volume. -Intensive color, flavor, and acidity of the bread by (excessive) fermentation.<br>-Higher specific volume and sensory scores by adding 15% germinated lupin flour.	Atudorei et al. 2022; Klupsaite et al. 2017; Villarino et al. 2015; Yaver and Bilgiçli 2021
Lupin	Protein isolate	5−10%	-Increased water absorption, dough development time, and stability. -Decreased dough elasticity.	-Reduced specific volume and increased hardness compared to wheat control.<br>-Lupin addition leading to green, earthy, malty, buttery and roasted aromas of wheat bread.	Paraskevopoulou et al. 2010, 2012
Pea	Flour	10−30%	-Increased water absorption<br>-Reduced dough stability.	-Above 10% replacement reducing loaf specific volume.<br>-Increased protein content.<br>-Toasting resulting in bread with comparable specific volume and loaf density compared to wheat control.	Millar et al. 2019; Mondor et al. 2014
Pea	Protein isolate and concentrate	5−15%	-Increased water absorption.<br>-Dough stability unaffected.	-Decrease loaf specific volume, crumb porosity and elasticity compared to wheat control.	Belc et al. 2021; Marchais et al. 2011
Red kidney bean	Flour	10−30%		-Doubled protein content.<br>-Decreased loaf specific volume and sensory scores for taste, flavor, and acceptability.	Bhol and Bosco 2014
Soy	Flour	5−18.5%	-Increased water absorption.	-Decreased specific volume.<br>-Higher specific volume and crumb softness by fermentation compared to wheat control.	Lazo-Velez et al. 2015; Huang et al. 2019; Wang et al., 2022c

Cowpea	Flour	5−20%	-Increased water absorption	-Increased protein, crude fiber and ash contents.<br>-Above 10% addition leading to reduced specific volume and denser structure, and decreased sensory scores for taste and acceptability.	Hallén et al. 2004; Olapade and Oluwole 2013
Mung bean	Flour	20%	-Increased water absorption, elastic modulus and viscous modulus.<br>-Decreased dough stability.		Meng et al. 2019
Jack bean	Flour and protein concentrate	10−20%		-Increased protein, crude fiber, fat, and ash contents.<br>-Decreased carbohydrate content.<br>-Comparable specific volume and sensory properties compared to control wheat bread.	Ugwuona and Suwaba 2013
Cereal side-streams					
Wheat bran	Protein isolate	12.2%		-Recognized for “high in protein” claim.<br>-Decreased specific volume and increased crumb hardness, and darker crumb compared to wheat control.	Arte et al. 2019
Rice bran	Protein concentrate	1−15%	-Reduced peak viscosity and increased pasting temperature.<br>-Decreased elastic and viscous moduli values.	-Increase bread protein, fiber, and total amino acid contents and higher radical scavenging activity.<br>-Reduced bread specific volume.<br>-Above 1% addition lowering the liking scores of color, taste, odor, texture, and overall liking.	Chinma et al. 2015; Jiamyangyuen et al. 2005
Oilseed side-streams					

Hemp seed cake	Flour	5−20%	-Decreased water absorption.<br>-Above 10% addition reducing dough stability, dough strength, and dough consistency.	-Reduced specific volume and increased crumb hardness with increasing levels of hemp cake flour.<br>-Darker crumb and crust compared to wheat control.	Pojić et al. 2015
Sunflower cake	Protein Isolate	1−9%		-Decreased specific volume and increased hardness.<br>-Increased protein and amino acid content with unpleasant taste and lower acceptability at the 9% addition level.	Mohammed et al. 2018
Flaxseed cake	Flour	5−10%		-Increased monounsaturated and polyunsaturated fatty acids, phenolic content, and antioxidant activity.<br>-10% substitution leading to negative sensory properties (flavor and texture).	Sanmartin et al. 2020
Rapeseed cake	Protein concentrate and isolate	10−20%		-Recognized for 'high in protein' claim.<br>-Reduced loaf specific volume and increased crumb hardness.<br>-Improved specific volume and softness, and nutritional quality (free amino acids and protein digestibility) by fermentation with in situ dextran production compared to wheat control.	Wang et al. 2022a
Minor cereals					
Sorghum	Flour	10−50%	-Increased water absorption.<br>-Decreased the dough stability and elasticity.	-Increased dietary fiber content, total phenolic compounds, and antioxidant activity.<br>-Reduced specific volume and increased crumb hardness.<br>-Improved bread texture quality and sensory properties i.e., reduced off-flavors such as bitter taste and aftertaste by fermentation with in situ dextran production.	Angioloni and Collar 2012; Jafari et al. 2017; Wang et al. 2020; Wu et al. 2018

				-Increased acceptability of sorghum-enriched bread by high hydrostatic pressure treatment	
Foxtail	Flour	20−50%		-Decreased glycemic index with foxtail addition.<br>-Increased sensory quality of foxtail bread by adding guar gum.	Das et al. 2021
Finger millet	Flour	10−30%	-Increased dough stability.<br>-Reduced dough energy, extensibility, and resistance to extension.	-Increased dietary fiber, phenolic acid content, and antioxidant activity.<br>-Reduced specific volume and increased crumb hardness.<br>-Increased loaf volume and decreased hardness due to increased α-amylase activity by hydrothermally treatment.<br>Extrusion treatment leading to higher specific volume and lower hardness, and higher sensory scores compared than un-extruded composite bread.	Mudau et al. 2021; Onyango et al. 2020; Patil et al. 2016
Tef	Flour	10−40%		-Tef addition resulting in higher levels of extractable polyphenols and anti-radical activity.<br>-Above 30% replacement leading to a decrease in specific volume and increase in crumb hardness.	Ronda et al. 2015
Proso millet	Flour	10−50%	-Above 20% replacement leading to reduced viscoelastic properties.	-Lower bread volume, firmer crumb and altered crumb pore structure.<br>-Improved texture with acceptable quality for as high as 50% replacement by adding emulsifier (DATEM and distilled monoglycerides) and enzymes (xylanase and transglutaminase).	Schoenlechner et al. 2013
Millets (finger, foxtail, barnyard, kodo, little, proso)	Flour	25%	-Decreased water absorption and dough stability.<br>-Increased starch gelatinization temperature and peak viscosity values.	-Increased dietary fiber content and resistant starch.<br>-Lower glycemic index.	Sharma et al. 2019
Barnyard millet	Flour	61.8%		-Textural quality of composite bread is comparable to wheat control by adding 6.8% gluten.	Singh et al. 2012

Pearl millet	Flour	50%	-Reduced water absorption and dough extensional properties, and increased dough stickiness.	-Decreased specific volume and increased crumb hardness.<br>-Increased specific volume and softness and delayed stalling rate by fermentation with in situ dextran production.<br>-Reduced starch digestibility and improved protein digestibility in vitro by fermentation.	Wang et al. 2019
Barley	Flour	40−60%	-Increased water absorption.<br>-Reduced dough stability and extensibility.	-Increased contents of fiber, total phenolic compounds, and antioxidant capacity.<br>-Decreased bread specific volume and increased crumb firmness, and lower sensory scores in taste compared to wheat control.<br>-Bread textural quality unaffected by fermentation.<br>-Decreased average Mw of β-glucan by fermentation.	Rieder et al. 2012; Robles-Ramírez et al. 2020
Barley	Middlings	15−60%	-Increased dough firmness.<br>-Decreased resistance to extension and dough elasticity.	-Increased contents of fiber and β-glucan.<br>-Lower loaf specific volume and negative textural properties above 30% addition.	Sullivan et al. 2011
Barley	β-glucan concentrate	2.5−10%	-Increased mixing time, dough stability and water absorption, and decreased dough extensibility above 5% replacement.		Ahmed 2015
Oat	Bran	5−40%	-Increased water absorption.<br>-Reduced dough stability, maximum resistance to extension, dough extensibility, and energy value.	-Increased contents of dietary fiber, the phenolic content, and antioxidant activity.<br>-Reduced specific volume and increased crumb hardness compared to wheat control.<br>-Bread quality unaffected by fermentation.<br>-Decreased β-glucan Mw by fermentation.	Rieder et al. 2012; Gamel et al. 2015; Saka et al. 2021

Oat	Flour	51%		-High levels of β-glucan (1.4−1.6 g /100 g fresh bread).<br>-Slight degradation (lower Mw) of β-glucan during proofing and baking.<br>-Content and Mw of oat β-glucan unaffected by fermentation.<br>-Bread volume and crumb properties mainly affected by gluten and water contents.	Flander et al. 2007, 2011
Oat	Soluble fiber	10−14%		-Lower specific volume and porosity, darker color, higher hardness, and lower springiness and cohesiveness compared to wheat control.<br>-The negative effects effectively counteracted by optimizing the water content in bread formulas.	Erive et al. 2020
Pseudocereals					
Quinoa	Flour	5−40%	-Decreased water absorption.<br>-Gluten secondary structure changed and gluten network diisrupted.	-Above 10% addition resulting in smaller specific volume, increased crumb hardness and coarse porosity.<br>-Higher antioxidant activity and reduced in vitro starch digestibility with lower estimated glycemic index due to higher contents of slowly digestible starch and resistant starch.<br>-Aroma and taste improved by 10-20% quinoa addition.<br>-Chemical, textural, and sensory features improved by fermentation.	Gostin 2019; Rizzello et al. 2016; Stikic et al. 2012; Wang et al. 2021; Xu et al. 2019
Buckwheat	Flour	15%		-Increased antioxidant activity.<br>-Lower specific volume but improved flavor and mouth feel compared to wheat bread.	Lin et al. 2009

Amaranth	Flour	25%		-Increased protein, lipid, fiber, ash, and phytate contents compared to control wheat bread.<br>-Up to 25% replacement providing bread with comparable specific volume and crumb softness to wheat control.	Miranda-Ramos et al. 2019; Sanz-Penella et al. 2013
Root and tuber crops					
Yam	Flour	5−75%	-Increased water absorption and decreased dough stability.<br>-Gluten network structure destroyed and contents of α-helix and β-sheet in gluten proteins reduced.	-Increased total phenolic content and radical scavenging capability.<br>-Decreased specific volume and increased crumb hardness, and lower overall acceptability.	Amandikwa et al. 2015; Li et al. 2020
Yam (purple)	Flour	10−50%		-Decreased contents of rapidly digestible starch and slowly digestible starch.	Liu et al. 2019
Cassava	Flour	10−50%	-Reduced water absorption, dough stability, and dough viscoelasticity.	-Decreased specific volume and increased crumb at or above 20% addition levels.<br>-Improved specific volume and reduced crumb hardness by dry-heat-moisture treatment.	Dudu et al. 2020; Jensen et al. 2015
Orange fleshed sweet potato (OFSP)	Flour	10−60%	-Decreased water absorption and increased dough development time.<br>-Decreased pasting temperature, peak viscosities, peak time, and dough stability.	-Breads containing 20% and 30% OFSP flour as an approach for the correcting vitamin A deficiency.<br>-Decreased loaf specific volume and increased hardness.	Chikpah et al. 2021; Edun et al. 2019; Nzamwita et al. 2017
Waste valorization					

Surplus bread	Bread-water slurry or hydrolysate	4.5−12.5%	-Reduced extensibility and dough level.	-Decreased specific volume and increased hardness.<br>-Improved specific volume and softness and increased microbial safety by fermentation with exopolysaccharide production.<br>-Enzymatic hydrolysis of starch in surplus bread hydrolysate with high-malto-oligosaccharides resulting in increased specific volume and reduced crumb hardness and staling rate compared to non-treated control.	Immonen et al. 2020, 2021
Brewers' spent grain (BSG)	Flour	5−20%	-Increased water absorption.<br>-Reduced dough stability.	-Decreased specific volume.<br>-Increased volume, preferrable aroma and taste traits and overall quality by fermentation of BSG (up to 10%).<br>-Adding fermented BSG at above 10% leading to reduced taste quality.<br>-Enzyme treatment leading to larger specific volume and softer crumb than extrusion treatment.	Plessas et al. 2007 Steinmacher et al. 2012; Vriesekoop et al. 2021
